# Challenging case of blunt vascular liver trauma: hematoma of the hepatic artery

**DOI:** 10.11604/pamj.2020.37.184.17786

**Published:** 2020-10-27

**Authors:** Pietro Fransvea, Gianluca Costa

**Affiliations:** 1 Surgical and Medical Department of Translational Medicine, Sant’Andrea Teaching Hospital, “Sapienza” University, Roma, Italy

**Keywords:** Blunt trauma, vascular injuries, hepatic artery

## Image in medicine

A 56-year-old male patient presented to emergency department after a car crash sustained a blunt thoracic and abdominal trauma. Patient according to the advanced trauma life support (A.T.L.S.®) protocol appeared stable. The patient complained of shortness of breathing and abdominal pain. Local examination revealed subcutaneous emphysema and hematoma of the right quadrant of the abdomen. Computed tomography angiography (CTA) of the abdomen revealed free fluid in the Morrison space and multiple contusion of the IV and V hepatic segment. The patient stared a conservative managment but due to a continue drop of haemoglobin, after 24h the patient was shifted to the operating room. At laparotomy a haematoma of the hepatic artery was found with clotted blood all around. Due to the absence of any signs of bleeding, no further procedure were done. The patient was discharged 15 postop with no complication. Despite the fact that treatment of liver injuries has dramatically evolved, severe liver traumas in polytraumatic patients still have a significant morbidity and mortality. The decision for an emergency laparotomy is usually taken based upon the presence of the “lethal triad”, consisting of coagulopathy, acidosis and hypothermia. In this case a continuous bleeding with platelet deplation was the reason to look into the abdomen with the suspect of Hollow viscus or mesenteric injuries.

**Figure 1:**
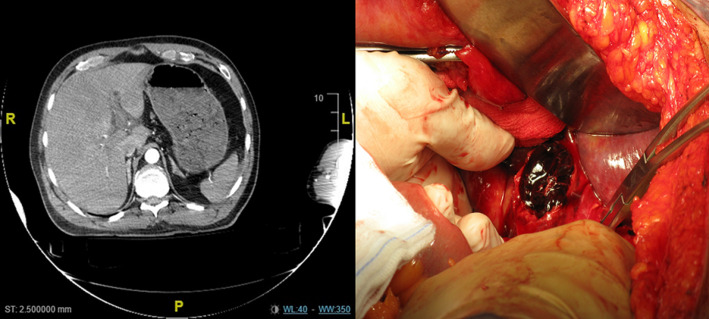
computed tomography angiography (CTA); multiple hepatic contusion, at laparotomy hematoma of the hepatic artery

